# The Technique of a Transplantation

**Published:** 1897-08

**Authors:** Louis Jack

**Affiliations:** Philadelphia


### THE TECHNIQUE OF A TRANSPLANTATION.

BY LOUIS JACK, D.D.S., PHILADELPHIA.

TRANSPLANTATION as introduced and practised by Dr. John Hunter was not attended by sufficient success to recommend it as one of the routine operations of dentistry. With the advent of antiseptic and aseptic surgery the attendant conditions and modifying factors involved in the operation have undergone such change that now the operation of transplantation may be viewed as a solution to hitherto insurmountable difficulties. For details of history and the general procedures, see article on "Plantation of

Teeth," in the proceedings of the Academy of Stomatology (International Dental Journal, March, 1896). The operation in the light of contemporary pathology has a well-defined technique, which is the purpose of the present demonstration to describe.

The patient, a boy aged nine and a half, had the left upper central incisor fractured transversely at about the middle of the crown a year previous to this date (May 29, 1897). The pulp was killed by the traumatism, probably by thrombosis of its vessels. At a later period an abscess developed, which received appropriate but partially effective treatment.

Noting the age of the patient and by exploration, it became a clear inference that the process of calcification of the tooth was incomplete, contraindicating an artificial crown as a permanent and lasting feature, the operation of transplantation was determined upon.

The first step of the operation consisted in obtaining reliable guides for the preparation of the teeth to be transplanted. A plaster impression of the upper labial teeth was taken, from which a model was made. By means of a probe passed into the pulp-canal the exact length of the canal was obtained and recorded.

The plaster tooth was cut away, leaving at the gingival margins the exact neck outlines, an excavation was then made in the plaster to the depth of the measurement recorded as the length of the root.

The excavation was intended to follow the neck outlines of the tooth carefully, and to leave an external labial lamina of plaster to represent the outer alveolar wall.

With this prepared model as a guide an appropriate tooth had to be selected from the stock of natural teeth, which are considerately saved by Dr. J. D. Thomas for this specific purpose.

It will be observed that, although the adjoining central incisor is of unusual form, a tooth was found which closely matched it, the anatomical correspondence being almost complete, so that no alteration was required in its length or outlines. The plaster teeth were varnished, the natural tooth set in its plaster alveolus, and retained by packing paper at any opening to secure it.

Two dies of Babbitt metal were made, and two counter-dies for the rough die are poured, one shallow, the other as deep as the entire crown length. An accurate tin pattern was made, a piece of eighteen-carat gold plate, No. 30, was well annealed, bent upon itself to cover the cutting-edges of the teeth, and was lightly malleted to rough adaptation with the die, avoiding striking the gold over the cutting-edges of the teeth, as it could be readily torn. Reannealed, the plate was set in the shallow counter-die, the die adjusted and swaged very lightly; both die and counter-die were covered with dampened tissue paper to avoid contamination of the gold. Reannealed and swaged between the perfect die and the counter-die.

The plate was formed to cover the lingual and labial faces of several of the adjacent teeth, including the transplanted one. The labial face of this tooth was permitted to be exposed at the upper half to reveal the gum-line and also slightly so on the lingual side for the same reason.

The plate was polished on the outer surfaces, but was left of dead finish on the inner side to afford better adhesion of the cement. It was also well to make a number of perforations at each end to increase the retention.

The pulp-canal of the tooth to be transplanted was opened throughout its length and reamed to almost the apex; it was then dropped in a three-per-cent. pyrozone solution, which disorganizes any decomposing organic matter present, and afterwards transferred to a one to two thousand solution of mercuric chloride. It should be remarked that any shreds of pericementum were scraped away as at least useless, and perhaps detrimental. After several hours immersion the tooth was removed from the sterilizing solution, its canal dried by means of wisps of bibulous paper, was filled with gutta-percha cones, packed tight; the opening upon the lingual face of the tooth was filled with gold. The tooth was returned to the bichloride solution, which, immediately before the operation, was heated to about 180° F.

All of the instruments used in the operation were kept in a three-per-cent. solution of hydronaphthol at a temperature of 200° F.

It was proposed to induce in the patient the initial stage of ether narcosis, that in which there is a transitory anæsthesia without loss of consciousness. The ether producing symptoms of nausea, local anæsthesia by refrigeration was substituted. A spray of ethyl chloride was directed against the gum until its edges were blanched, when the gum was detached from the neck of the tooth by means of a lancet, and the tooth immediately extracted. The mouth was then freely sprayed with the hydronaphthol solution, which was also used prior to the extraction. After bleeding was lessened the same solution of hydronaphthol was blown from an atomizer under the force of compressed air into the alveolus, this was continued for fully a minute.

The next step consisted of the partial decalcification of the cementum. The process was not carried to the full extent described and recommended by Dr. Amoedo. An opening was made in a piece of rubber dam through which the root of the tooth was thrust to the enamel margins; the loose ends of the dam were brought over the crown of the tooth enclosing it, and protecting the enamel from the action of the ten-per-cent. solution of hydrochloric acid, in which the root was now immersed.

This removed any microscopic deposits of calculi which might be present upon the root, the superficial cementum was also slightly softened, forming the safest and most satisfactory scaffolding upon which the attaching tissue might be built.

The acid was neutralized by dipping the tooth in dilute ammonia water.

The tampon of cotton, saturated with the hydronaphthol solution, was removed from the alveolus, and the tooth inserted to test its position. The retaining cup was removed from the antiseptic solution and set in position, for assurance of accuracy of adaptation.

The alveolus and teeth were again sprayed with the forcible jet of hydronaphthol, napkins were placed in position, and the parts dried. A thin mix of zinc phosphate was made; the tooth for replantation was set in its alveolus, the cement was placed in the retaining appliance which was then quickly pressed into position, and held firmly until the cement was hardened.

The appliance is to be worn for six months, as absolute immobility is a *sine qua non* for the success of all of these plantation operations.

The above procedures of the stated case were carried out in the presence of the council and some other members of the Academy.—*Reported by* Henry H. Burchard, *Editor Academy of Stomatology.*

